# Supplementary material for: Patterns of Intron Gain and Loss in Fungi
Source: PLoS Biol. 2004 Nov 30;2(12):e422. doi: 10.1371/journal.pbio.0020422 (PMC532390; doi:10.1371/journal.pbio.0020422)
Supplement: Table S1 — Also available at http://genes.mit.edu/NielsenEtAl/. (4.3 MB ZIP). [file pbio.0020422.st001.zip › NielsenEtAl/html/1222.html]

AN2926.1.NCU00981.1.MG10835.1.FG01135.1


```
 CLUSTAL W (1.82) Multiple Sequence Alignments - Introns Inserted


Sequence 1: AN2926.1	282 aa
Sequence 2: FG01135.1	260 aa
Sequence 3: NCU00981.1	261 aa
Sequence 4: MG10835.1	260 aa
Alignment Length: 283 aa
Number Identitical Residues: 166 aa
Alignment Score (without introns) 7392


MG10835.1 	M0PQNE~YIE~RWRKQHG2QRLDHEERARKKAAREEGHGKSAKAQNLRGLKAKLFAQERR
NCU00981.1	M0PQNE~YIE1RAQKLHG~KRLDTEERARKKAAR-EGHKQSENAQNLRGLRAKLFAKERH
FG01135.1 	M0PQNE~YME~RWRKLHG~RRLDHEERVRKRTAR-EGHKASQDAQNLRGLRAKLYQQRRH
AN2926.1  	M0PQNE2YIE~RWTKQHG~KRLDHDERVRKREAR-QSHQQSKDAQNLRGLRAKLYQQKRH
          	* **** *:* *  * ** :*** :**.**: ** :.*  * .*******:***: :.*:

MG10835.1 	KEKIRMKKAIKQHEERNVKGAAADKDPTNPVPAYLLDRNTPNTSKA2LS~SSIKQARAEK
NCU00981.1	AQKIQMRKAIKQHEERNVKGAPEEKDPSNPVPAYLLDRSNPTSAKA~LS2SQIKSKRAEK
FG01135.1 	KEKIQMKKQIKAHEERNVKTADEKDPT-TPMPSYLLDRTNPSTAKA~LS~SAIKNKRAEK
AN2926.1  	AEKIQMRKRIKAQEEKNVKSSAPSEPSKTPLPQYLLDRSEATNAKA~LS~SAIKDKRAEK
          	 :**:*:* ** :**:*** :  .. ...*:* *****. ...:** ** * **. ****

MG10835.1 	AAKFSVPIPKVRGISEEELFKVVHTGK-SKRKSWKRMVTKPTFVGQNFTRR2NPKYERFI
NCU00981.1	AARFSVPIPKVKGISEEELFKVVKTGKKVHKKGWKRVVTKPTFVGPDFTRR~PVKYERFI
FG01135.1 	AAKFAVPLPKVRGISEEEMFKVVKTGKKIQKKAWKRMVTKPTFVGPDFTRR~NPKHERFI
AN2926.1  	AAKFAVPLPKVKGISEEEMFKVVNTGKKTHKKSWKRMITKPTFVGNDFTRR~PVKYERFI
          	**:*:**:***:******:****:***. ::*.***::******* :****   *:****

MG10835.1 	R~PMGLRYKKAHVTH---------------------PE0LNVTVHLPILGV1KKNPSNPL
NCU00981.1	R~PMGLRYKKANVTH---------------------PT~LNVTVQLPILGV~KKNPSNPL
FG01135.1 	R~PMGLRYKKANVTH---------------------PE~LGVTVQLPIISV~KKNPQNPL
AN2926.1  	R2PMGLRYKKANVTQYVLSLATIGVYLGFTDKFSCSPE~MAVTVQLPILSV~KKNPQNPL
          	* *********:**:   : :: .   . :.. :.:*  : ***:***:.* ****.***

MG10835.1 	Y2TQLGVLSKGTVIEVNVSELGIMAGN-KVAWGRYAQVTNSP2E~NDGCVNA~VLLV
NCU00981.1	Y~TQLGVLSKGTIIEVNVSDLGMVTASGKIAWGRYAQITNNP~E0NDGCLNA~VLLV
FG01135.1 	Y~TQLGVLTKGTVIEVNVSELGLVTAGGKVVWGRYAQVTNNP~E~NEGCINS~VLLV
AN2926.1  	Y~TQLGVLTKGTVIEVNVSELGIVTAGGKVAWGKYAQITNTP~E~NDGCVNA2VLLV
          	* ******:***:******:**:::...*:.**:***:**.* * *:**:*: ****
```
